# Symmetrical pH Electrochemical Cell Coupled to Constant Potential Coulometry for Improved Sensitivity and Precision: Part 2. Submersible Probe for In Situ Measurements

**DOI:** 10.1021/acsmeasuresciau.5c00198

**Published:** 2026-02-10

**Authors:** Robin Nussbaum, Stéphane Jeanneret, Thomas Cherubini, Mary-Lou Tercier-Waeber, Dario Omanović, Eric Bakker

**Affiliations:** † Department of Inorganic and Analytical Chemistry, 30500 University of Geneva, Quai Ernest-Ansermet 30, 1211 Geneva,Switzerland; ‡ Laboratory for Physical Chemistry of Traces. Center for Marine and Environmental Research, 54583 Ruđer Bošković Institute, POB 180, 10002 Zagreb, Croatia

**Keywords:** constant potential coulometry, high sensitivity, pH glass electrode, seawater pH, submersible probe

## Abstract

Seawater pH is a critical parameter influencing many processes in the ocean. Today it is mainly measured by indicator-based spectrophotometry to allow for high precision. This, however, is at the expense of traceability and systematic errors originating from changes in temperature, salinity, and other matrix effects. Moreover, in routine practice, this approach is not performed in situ and requires sampling and manual manipulations, which are prone to introduce additional errors, including gas exchange with the atmosphere. Unfortunately, in the last few decades, the electrochemical sensing community has failed to make efforts to improve the performance of the main method, which is potentiometric detection with pH glass electrodes. To address this, we aim here to improve the sensitivity and precision of submersible pH probes on the basis of pH glass electrodes by minimizing systematic errors from temperature changes and by implementing a recently described coulometric method. The electrodes are mounted in a symmetrical cell reported in part 1 of this work to reduce sensor drift and minimize inaccuracies due to liquid junction potential variations and pH changes of the inner solution from temperature fluctuations. The development and construction of the probe are explained. The circuit is evaluated, and the sensors are calibrated over a range of temperatures, approaching ideal behavior. The submersible probe was deployed in situ in April 2025 in the vertically stratified Krka River Estuary in Croatia. The precision of the probe was evaluated in situ by stability experiments in the seawater layer. The determined precision is 0.001 pH unit, which is significantly better than that reported earlier for routine pH probes. A recalibration procedure with synthetic seawater is also evaluated for minimizing drift. A depth profile with a changing salinity was performed and compared with multiparameter probes.

## Graphical abstract

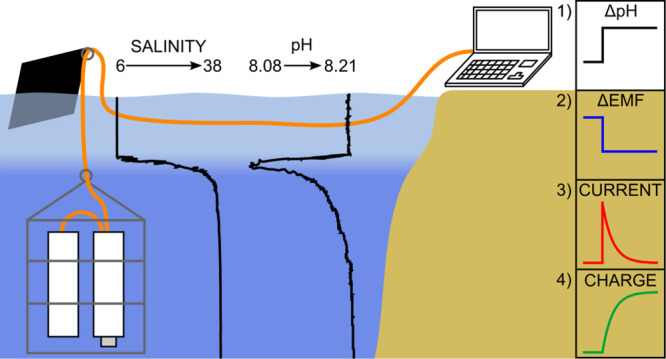

## Introduction

The level of carbon dioxide (CO_2_) in the atmosphere has risen by nearly 40% compared with preindustrial values.^1^ Up to one-third of atmospheric CO_2_ dissolves in the ocean and alters the seawater pH by a process referred to as *ocean acidification* ^2^ since seawater pH is governed by carbonate chemistry.^3^ When CO_2_ dissolves, it reacts with water to form carbonic acid, which then dissociates into bicarbonate and hydrogen ions. These changes in carbonate equilibrium affect various important processes such as marine calcification and metal speciation.^4–6^ It is therefore crucial to closely monitor seawater pH. Ocean acidification lowered seawater pH by around 0.1 since preindustrial times and is expected to decrease by 0.3–0.4 units more until 2100.^7,8^ Studies in the Northwest, Northeast Atlantic and central North Pacific demonstrated similar long-term surface pH decrease rates of about −0.002 pH year^–1^.^9–11^ These minute changes require highly precise measurements.^12^

The most common analytical method for seawater pH relies on spectrophotometric measurement using metacresol purple with a reported precision better than 0.001 pH unit with the pH being expressed using the total hydrogen ion concentration scale (pH_T_).^13,14^ Methodologies to perform automated measurements in the laboratory have been reported.^15^ The main drawback of this approach is that sampling is required, making it incompatible with highly time-resolved measurements over a long time period. While spectrophotometric measurements have been successfully implemented in submersible devices,^16–18^ routine pH measurements used to report ocean acidification are still performed manually by adding dye to a cuvette at 25 °C after sampling, which seems inadequate given the importance of this parameter and the risk of changing the pH by gas exchange with the atmosphere. Even submersible systems come with their limitations that include a frequent need of blank measurement and the introduction of indicator dye to the seawater sample.^16^ On the electrochemical side, efforts have been made to measure seawater pH in situ using different techniques. Potentiometric pH sensors based on the Honeywell ion-sensitive field effect transistors (ISFETs) were successfully used to develop submersible pH sensors.^12,19^ They were deployed in the open sea but also demonstrated attractive performance in dynamic environments such as estuaries.^20–22^

Potentiometry with glass electrodes remains the main method for pH measurement in many fields of research.^23^ Unfortunately, they have been largely abandoned for seawater pH measurements because of inadequacies of commercially available probe designs and instrumentation that exhibit signal drift and inaccuracies caused by liquid junction potential variations with changing ionic strength.^14^ To some extent, the electrochemical sensor research community is to blame for this situation. It was decided many years ago that this measurement technology is now mature and has therefore not sufficiently invested to make these sensors fit for purpose.

The following considerations can be made to improve the sensitivity and precision of potentiometric pH probes. The liquid junction of routine pH probes is often fabricated from porous ceramic materials that are prone to clogging and ingress of seawater into the reference electrode (RE) compartment, which results in undesired potential variations and drift. Research by Covington et al. showed that an open-junction design provides much smaller liquid junction potential variations, even in estuarine waters.^24–26^ Junction-free REs were also investigated for ISFET-based sensors to try to solve this issue.^12^ Another source of error is the influence of temperature on the inner buffer solution of potentiometric pH probes, which are designed to provide a zero-point pH of 7, independent of temperature. However, inner solution buffers are never ideal and exhibit residual potential changes on the order of 1–2 mV (or 20–30 mpH), which is excessive in the context of seawater pH measurements. To overcome these chemical limitations, our group described in part 1 of this work a symmetrical pH electrochemical cell with an open-junction design that incorporates two equally built pH glass electrodes.^27^ This allows one to fully compensate for the influence of the inner solution pH with temperature and should minimize the change in liquid junction potential with sample salinity. However, it does not remove the uncertainty resulting from junction potential change when changing from NIST buffers to seawater which is on the order of a few mV.^28^ The uncertainty depends, however, on the type of junction and should be studied for each design for proper uncertainty assessment.

Moreover, glass electrodes remain limited by their Nernstian sensitivity. About a decade ago, a novel readout called constant potential coulometry was introduced for solid-contact electrodes to overcome this intrinsic limited sensitivity.^29^ This alternative readout took advantage of the capacitive nature of the transducing layer and could thus only be used for solid-contact electrodes.^30^ In this protocol, a constant potential is imposed between the ion-selective electrode (ISE) and the RE. Therefore, any potential change at the ISE induces an opposite potential change on the transducing layer, giving rise to a transient current. The latter is integrated to obtain the charge that obeys the following relationship.^31^

$$Q=C\frac{s}{z_{i}}\log\;\frac{a_{i}(\mathrm{initial})}{a_{i}(\mathrm{final})}=Cs\mathrm{\Delta pH}$$ 1

where *Q* is the charge, *C* is the capacitance, *s* is the Nernstian slope for a monovalent ion, *z*_*i*_ is the charge of said ion, and *a*_*i*_ is its activity. Our group improved on this method by replacing the chemical capacitance by an electronic one,^32^ introducing electronic control,^33^ and widening the scope of electrodes to highly resistive membranes such as pH glass electrodes by implementing a high-impedance voltage follower.^34^ The latter marked a major breakthrough by completely separating the current measurement from the electrochemical cell. This now allows for the use of glass electrodes with a coulometric readout.

In part 1 of this work, we presented a symmetrical flow cell with open liquid junctions.^27^ A novel electrochemical setup for constant potential coulometry was introduced and showed attractive signal repeatability and precision. That work was, however, still based on a benchtop instrument and not yet applicable for in situ measurements.

Based on all these advances, we present here a novel pH submersible probe capable of both potentiometric and coulometric readout. The sensing head, composed of two glass electrodes embedded in a symmetrical cell with two dilution junctions, was described in part 1.^27^ The probe also includes a recalibration procedure with synthetic seawater of a known pH. The characteristics of the probe are described. The electronic circuit is characterized to ensure a behavior close to theoretical expectations. The influence of temperature on the sensor signal is assessed in the laboratory to correct for in situ temperature fluctuations. As a field application, the probe was deployed in April 2025 in the Krka River Estuary, Croatia. Both signal repeatability and precision were evaluated. The data were compared with commercially available submersible multiparameter probes with an integrated potentiometric pH measurement.

## Methods

### Materials and Sensors

Disodium hydrogen phosphate (Na_2_HPO_4_, >99%), potassium chloride (KCl, >99.5%), potassium dihydrogen phosphate (KH_2_PO_4_, >99%), sodium chloride (NaCl, >99%), sodium sulfate (Na_2_SO_4_), tris­(hydroxymethyl)­aminomethane (Tris) were purchased from Sigma-Aldrich, Merck, Germany. Volumetric 1 M hydrochloric acid (HCl) was purchased from Fisher Chemicals. Magnesium chloride (MgCl_2_) and calcium chloride (CaCl_2_) 1 M volumetric solutions were purchased from Honeywell Fluka, Fischer Scientific, Switzerland. NIST/DIN buffer solutions of phthalate (pH = 4.008), phosphate (pH = 6.865), and borax (pH = 9.180) were purchased from Mettler Toledo, Switzerland. Ag/AgCl-coated wires were purchased from Metrohm, Switzerland. Nafion tubing TT-060 was purchased from Perma Pure, USA. Idex Nonmetallic Inline Check Valves were purchased from Cole-Parmer, U.K. Submersible cables, connectors, and cable storage reel were purchased from Novasub, Seascape Subsea, The Netherlands. NKP-DE-B06B peristatic pumps were purchased from Kamoer Fluid Tech, China. A NIST phosphate buffer was prepared in house according to published composition.^35^ The precise volumes of volumetric NaOH solution required to prepare NIST phophate buffer solutions with altered pH were calculated with the Henderson–Hasselbalch equation. The synthetic seawater was prepared following a published procedure.^36^

The submersible multiparameter probes used in this work are an EXO2 Probe (YSI, Xylem Inc., USA) and an OS316-*Plus* (Idronaut, Italy). The salinity values are obtained from conductivity measurements. The Ag/AgCl common reference elements were prepared as in the first part of this work. The pH glass electrodes were generously supplied by Metroglas AG, Switzerland. They are made of robust glass with a long lifetime, analogous to the ones sold to Idronaut, Italy, for implementation in their submersible multiparameter probes. Their impedance was determined to be around 50 MΩ. The temperature probe was a Pt1000 sensor embedded in a metallic tube.

### Probe Design

The entire probe, the *CouloProbe*, was built and designed in house and is shown in Figure 1a and Supporting Information. The two casings were made of Delrin tubes (600 mm length, 100 mm outer diameter, and 80 mm inner diameter purchased from Amsler & Frey AG, Switzerland) closed by transparent lids made of poly­(methyl methacrylate) (PMMA). The water tightness is ensured by nitrile butadiene rubber (NBR) O-rings inserted in the side of the caps. The main casing contains the electronics and a fluidic part with the sensors. Both parts are separated by a watertight partition PMMA wall in case of leakage from the sensing head. The second casing contains 5 NKP peristaltic pumps running at 5.5 V that are mounted on a metallic rack and connected to the main casing by a submersible cable. Both casings are mounted to a titanium cage to which the reagents and waste bags are fixed. Diving weights are attached to the bottom of the cage to compensate for the air volume inside of the instrument. The sensing head was presented in part 1 (Figure 1 of that work).^27^ It contains a glass electrode in the sample compartment, a glass electrode in the reference compartment, and a common reference element in the middle channel that is filled with 3 M KCl solution. The reference glass electrode is, except when stated otherwise, immersed in a NIST borax buffer. The sample glass electrode can be in contact with NIST borax for the reference measurement, seawater for sample analysis, and synthetic seawater for recalibration. Two check valves are added after the exits to prevent backflow from the waste bags. A heat-exchange coil was added before the pH reference compartment to ensure that the reference buffer was measured at the same temperature as the sample.

**Figure 1:**
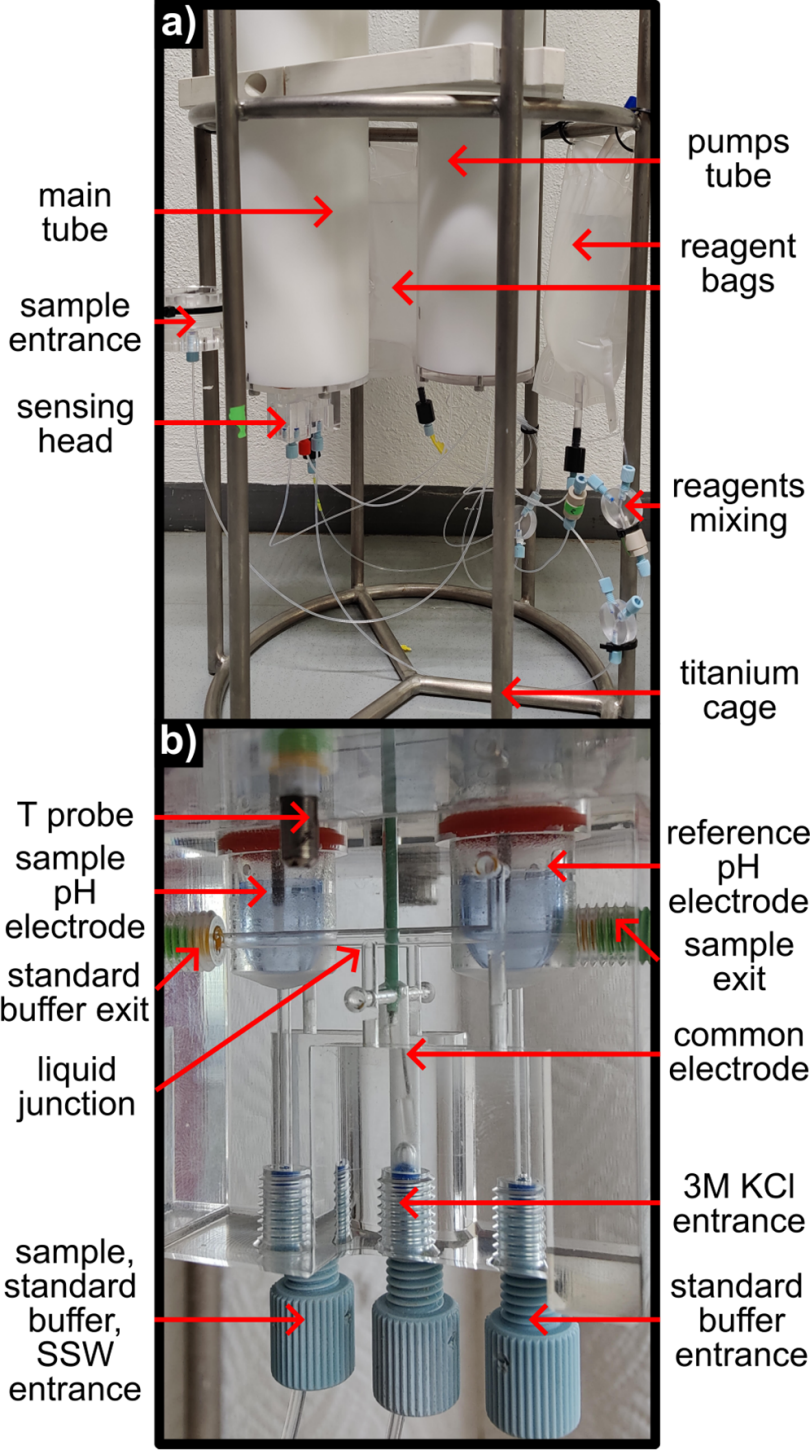
(a) Picture of the Couloprobe. (b) Zoomed-in picture of the sensing head.

### Electrochemical Field Equipment

In our previous work on constant potential coulometry, a homemade electronic circuit was added in between a commercial potentiostat and the electrochemical cell.^34,37^ For this work, a submersible instrument was developed for the first time to perform *in situ* measurements (Figure 2). It is a complete automated potentiometric and coulometric pH measurement device that can read user-written methods, execute them to perform the measurements, record and save the results, and send them to the user through a cabled communication link. Pictures of the electronic board are shown in Figure S1, and the detailed electronic schemes are given in Figures S9–S15. The elements were sized to fit inside a tube with an inner diameter of 80 mm. The heart of the instrument is a homemade board (Figure 2, top part) that contains a microcontroller chip (STM32L4R5) running a real-time operating system called Chibios. It has 640kB of internal memory that is used for the rapid capacitive measurements. The following parts are added: an external microSD card to save the measurement data, an external EEPROM memory to save the methods and settings, a communication link through an isolated RS485 full duplex link (ISOW1432), and an extension for the control of the pumps. (Driver: TB6612, PWM: PCA9635, and control: MCP23017). The power is supplied by a 12 V battery through a communication cable. The temperature is measured using a PT1000 connected to a MAX31865 chip.

**Figure 2:**
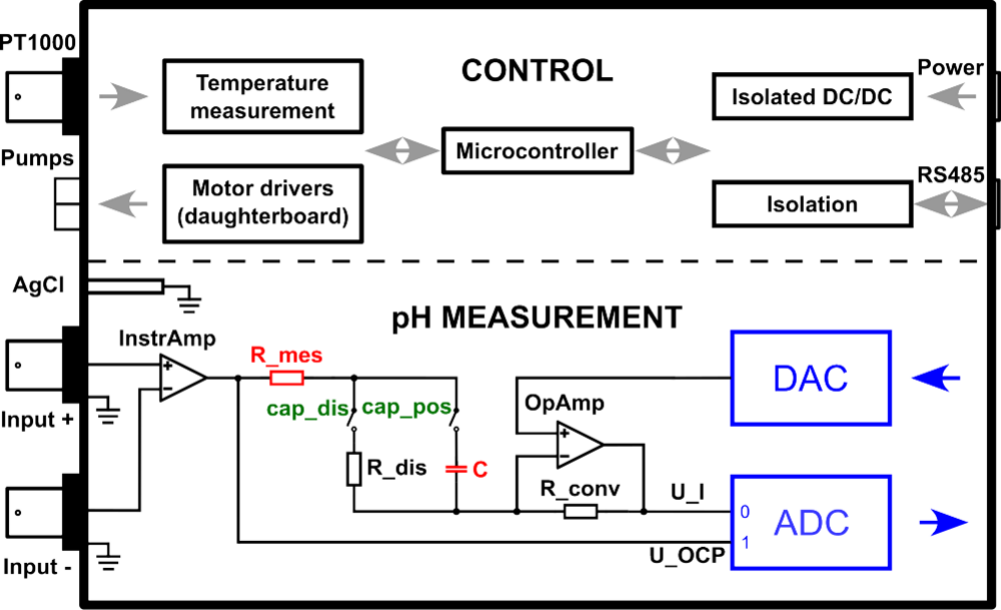
Electronic circuit developed for this project. The top part represents the control part with power, pump control, and temperature measurement. The bottom part is the pH measurement circuit with the measuring RC components (red) and relays (green). The sample glass electrode was plugged on the positive input, while the reference one was connected on the negative input.

The pH measurement circuit (Figure 2, bottom part) is designed to handle different possibilities through user-written methods. It consists of a 20-bit DAC (DAC1220), one 24-bit ADC (ADS1255), reed relays (COTO9007 and HE721C0500), and adaptation circuits. The DAC used in this instrument is very precise, but due to a necessary hardware reconfiguration during the development phase, the calibration of the current measurement could not be done optimally, which resulted in a residual constant offset. However, it does not have consequences on the coulometric signal, because it is constant over the whole measurement series and subtracted from the sample measurements. The analog input stage (instrumentation amplifier) is an AD8220 chip that is capable of measuring the difference between two points with a current as low as 25 pA. The current to voltage conversion of the measurement by the instrument is performed using a standard operational amplifier (OPA192) with a resistor.

Potentiometric measurements are performed by averaging the potential signals over consecutive periods of 3 s. The coulometric measurements are performed through a polarized tantalum capacitor of 438 μF and a 1 kΩ resistor (both indicated in red in Figure 3, bottom part). The RC time constant of the circuit is about 0.44 s. Thus, the transient currents are recorded for 2.5 s (>5 RC) to ensure almost complete charging of the capacitor.

**Figure 3:**
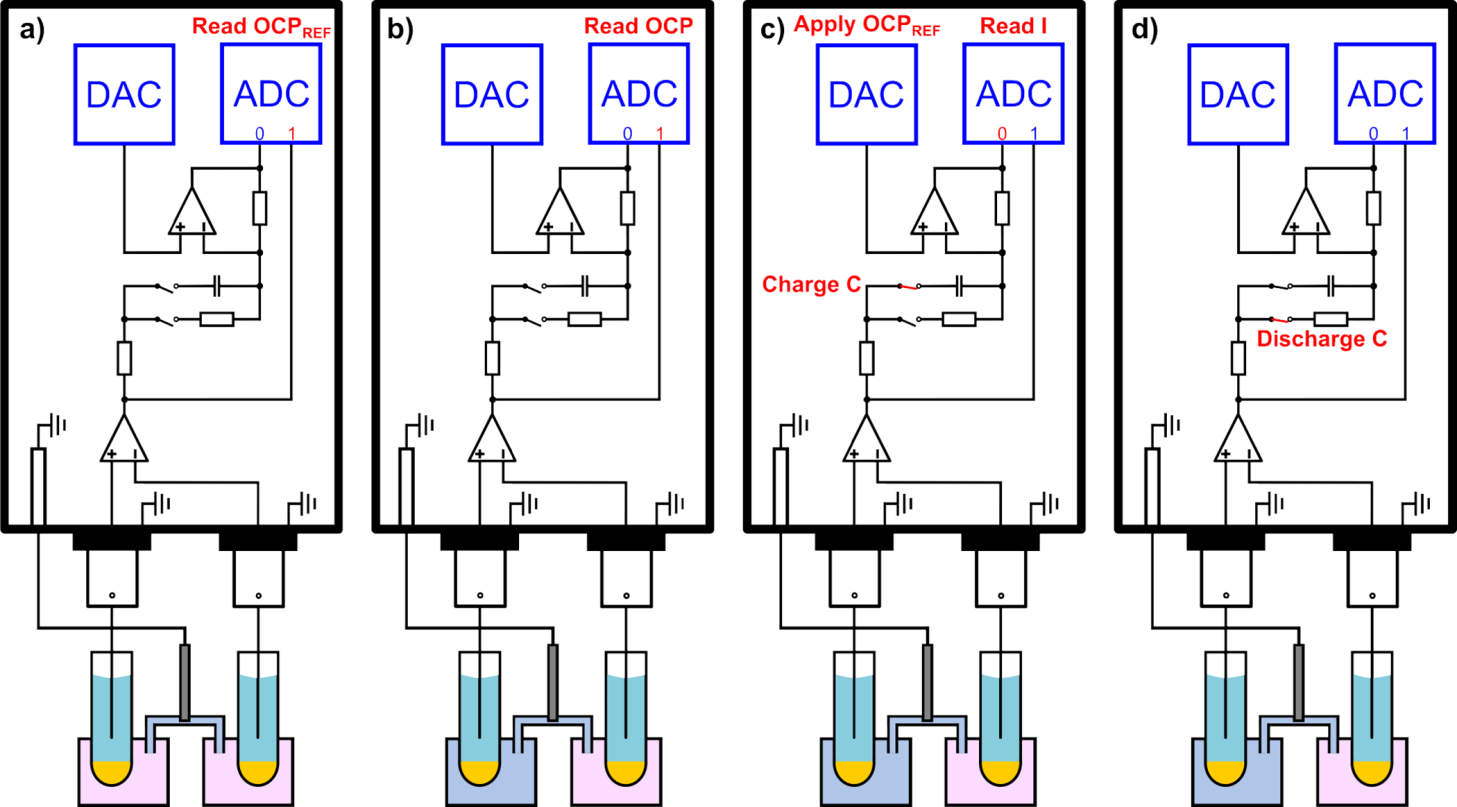
Experimental measurement procedure. (a) OCP with both glass electrodes in borax buffer is recorded. (b) Sample is introduced, and the OCP is recorded. (c) OCP ref is enforced by the instrument, and the transient current is recorded. (d) Capacitor is short circuited and discharged. One can then go back to step (b).

### Measurement Principle

Before each measurement, the temperature is recorded. The measurement routine for each sample is depicted in Figure 3. The first step is to record the open circuit potential (OCP) when both the sample and reference compartments are filled with the NIST borax buffer (Figure 3a). This step allows one to consider the experimental deviation of the symmetrical OCP from the theoretical 0 mV and to evaluate the residual charge induced by the calibration of the transient measurement current in a coulometric mode. The reference signal for both potentiometry and coulometry is then subtracted from all sample measurements. Subsequently, the sample is continuously pumped into the cell for 120 s. The reference buffer and 3 M KCl solutions are flowed during the last 30 s to ensure buffer renewal in the reference compartment and reproducible liquid junction potentials at the two open junctions. The OCP is then allowed to stabilize for 300 s and subsequently recorded for 3 s (Figure 3b). This completes the potentiometric measurement. The DAC is then used to impose the reference OCP, the capacitor is connected, and the transient current over the capacitor is recorded by the ADC (Figure 3c). The charge is obtained by integrating the current with Python using the SciPy integrate cumulative trapezoid method. This is the coulometric signal. The capacitor is then short-circuited through a resistor to prepare it for the next measurement (Figure 3d). One may then perform multiple potentiometric and coulometric measurements in the same sample to obtain information on signal repeatability, which refers to the standard deviation between measurements carried out in the same sample. In this work, three potentiometric and coulometric measurements were performed for each sample. The average signal repeatability refers to the average of these signal repeatabilities for different samples. On the other hand, precision is defined as the standard deviation of the pH values obtained for repeatedly measured samples of which the composition is assumed to remain constant.

### Data Analysis Procedure

In the experimental setup chosen for this work, to convert the potential difference or the charge to a pH value, one requires knowledge of the temperature-dependent slope and the zero-point pH, which is a function of the known reference NIST buffer with temperature. The OCP and charge are converted to pH using eqs 2 and 3.

$${\mathrm{pH}}_{E}=\frac{\Delta E}{s_{E}(T)}+{\mathrm{pH}}_{E=0}(T)$$ 2

$${\mathrm{pH}}_{Q}=\frac{Q}{s_{Q}(T)}+{\mathrm{pH}}_{Q=0}(T)$$ 3

The data processing was done with scripts in Wolfram Mathematica 14.2, and the parameters were obtained as described in a following part. As the temperature influences both the slope and the zero-point pH, its measurement error (the standard deviation over 30 temperature measurements) was considered during the conversion of the signal repeatability from potential difference or charge unit to pH unit. The signal repeatability conversion procedure is explained in the SI on page S4 with charge as an example.

### Site of Study

The Krka river is located on the eastern coast of the Adriatic Sea in Croatia. After a series of large waterfalls, the 23 km long Krka River Estuary begins. As with many Mediterranean estuaries, the tides have a weak amplitude, which induces permanent vertical stratification.^38^ The Ruder Bošković Institute, Center for Marine and Environmental Research, Zagreb, Croatia operates a marine station located in this estuary with an on-shore laboratory (43.73863, 15.87582). All in situ studies were performed 40 m offshore this site. The submersible probe was deployed in water and attached to a moored buoy to perform autonomous measurements at given depths.

## Results and Discussion

### Characterization of the Electronic Circuit

The behavior of the electronic circuit was investigated to ensure appropriate behavior of the instrument. An external source meter was first used to impose a voltage on the electronics of the probe. The potential and current were recorded 3 times for each external voltage. When imposing 0 mV, the recorded potential was 55 μV. This potential was then enforced by the probe’s electronics, and the input voltage was varied in 0.6 mV steps, corresponding to 0.01 pH changes at 25 °C (Figure S2a,b). One may notice that the charge was 1.1 μC instead of 0 μC when the external voltage was 0 mV. It is referred to as *charge offset* in the text. This is due to the current calibration step of the instrument explained above and not the OCP offset of 55 μV which would result in a charge offset of only 0.024 μC. A theoretical fit was performed by multiplying the voltage by the capacitance value to obtain the charge and adding the charge offset to each point (Figure S2b, blue line). The maximum deviation between experimental data and theory was 0.017 μC with error increasing when the charge was too close to 0. This increased error with decreasing peak height is explained by the current noise. This limitation is likely unimportant during real-world experiments, as the sample and calibration solution pH are typically more than half a unit (corresponding to >30 mV) from the reference buffer in an aquatic system measurement.

The behavior of the circuit was then evaluated for chloride sensing as a chemical model system. An Ag/AgCl electrode was used as the sensing electrode, an Ag/AgCl/3 M KCl/1 M LiOAc as the RE, and an Ag/AgCl wire as the common reference element. The starting chloride concentration was 1 mM, which was successively increased to 100 mM (Figure S3). During this experiment, the connections for the sensing and reference electrodes were switched to place the highest impedance electrode at the sample input. Because of this, the chloride potential response exhibits an opposite sign compared with the expected one from the Nernst equation. The slope was slightly sub-Nernstian (−24.3 μC, corresponding to −55.5 mV), as previously observed with Ag/AgCl electrodes.^34^ The capacitive currents were recorded and fit with an RC exponential decay of the circuit and the recorded OCP (Figure 4a). The fit to the calculated charges from the OCP values was again excellent with deviations from theory below 5%.

**Figure 4:**
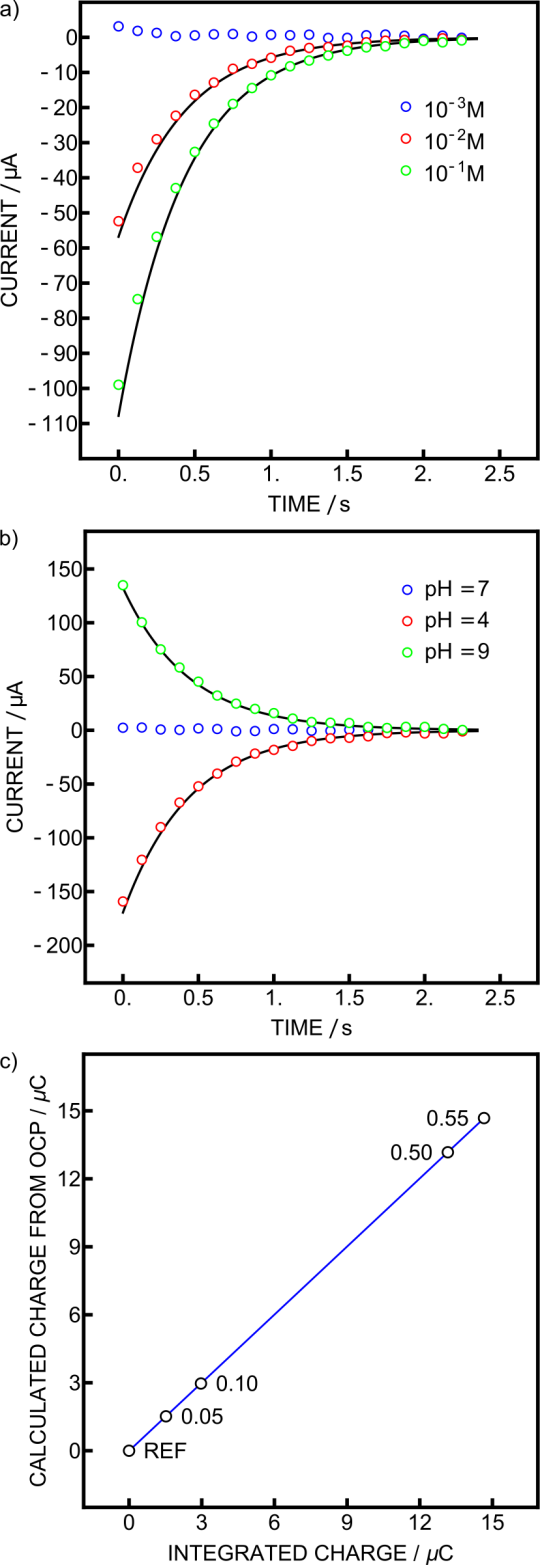
(a) Currents obtained with Ag/AgCl electrodes in different NaCl concentrations with theoretical fit in black. (b) Currents observed during pH calibration with phosphate as reference buffer with theoretical fits in black. (c) Correlation plot between charges obtained from coulometry (*x*-axis) and calculated from potentiometry (*y*-axis).

The circuit was then evaluated in the flow cell with two glass electrodes measured against a common reference element, as explained in the experimental part. The solution used for the pH electrode acting as a reference was a NIST phosphate buffer for which the temperature dependence of the pH is known. The OCP with the same solution in the sample compartment was −1.5 mV. Ideally, it should be zero, but as discussed in part 1 of this work, glass electrodes are never exactly identical.^27^ However, this can be easily overcome, as the OCP and charge measured in the reference solution are subtracted from all following measurements. This experiment was first performed at 20 °C, giving an electrode slope of 25.4 μC (58.0 mV), less than 1% away from theory, with excellent linearity (Figure S4). The current was again fitted as described above (Figure 4b), giving deviations from the theory below 2%. A NIST phosphate buffer prepared in the laboratory was then used to investigate small pH changes. The following pH changes were evaluated: 0.05, 0.1, 0.5, and 0.55 (Figure 4c) at 25 °C. The potentiometric slope was found to be slightly super-Nernstian (−59.9 mV, 1% deviation). The coulometric slope was 26.2 μC, corresponding to 59.8 mV or 99% of the potentiometric one, showing that the correlation between OCP and integrated charge is excellent. Moreover, the current fits are again excellent with charges deviating from theory below 0.5%. However, this deviation, due to the tolerance of the electric components, can have a significant influence on the pH value. For example, for a difference of 1 pH unit, which is on the order expected between seawater and the reference solution, a deviation of only 0.2% would result in a 0.002 pH unit between both readouts. It is therefore crucial to calibrate them individually rather than convert one into the other.

### Experimental Parameter Determination and Stability Experiment in the Laboratory

When conducting pH measurements, the temperature changes both the slope of the glass electrode response and the pH of the calibration buffers.^35^ Previous work on constant potential coulometry was performed in a temperature-controlled environment to avoid unexpected fluctuations, especially of sample pH.^34,37^ However, the temperature in situ will fluctuate, and the sensor will need to operate accurately in the range of temperatures that are expected in the field, in this case between 10 and 20 °C.

To account for nonidealities of the system, the experimental slopes and behavior of the NIST borax buffer were determined by immersing the flow cell in a thermostated bath. NIST borax and phosphate were successively flowed through the sample compartment at 25, 20, 15, 10, and 5 °C. The corresponding experimental slopes for potentiometry and coulometry are presented in Figure 5. They were determined using the signal readout and the pH values reported by the manufacturer for each temperature. As the point at 25 °C was slightly off and no such temperature was experienced during the field application, it was excluded from the plot and data analysis procedure. Both techniques produced excellent results, as the deviation of the experimental slope from Nernstian behavior was at most 0.6%. The experimental temperature-dependent pH of the NIST borax buffer was obtained from the experimental zero-point pH at different temperatures (Figure S5). For both potentiometry and coulometry, the values were in the accuracy range reported by the manufacturer (±0.015 pH unit) with maximum deviations being 0.010 and 0.012 pH units, respectively. This experiment allows one to build for both readouts a linear correction function for the slope as a function of the temperature. Due to the limited temperature range encountered in this work, a quadratic correction function for the borax reference pH was preferred over the fit function described by Bates.^35^ The parameters of the equations are shown in Table S1, and the conversion from signal to sample pH could now be obtained using eqs 2 and 3. The precisions obtained during this experiment were 0.2 and 0.3 mpH for potentiometry and coulometry.

**Figure 5:**
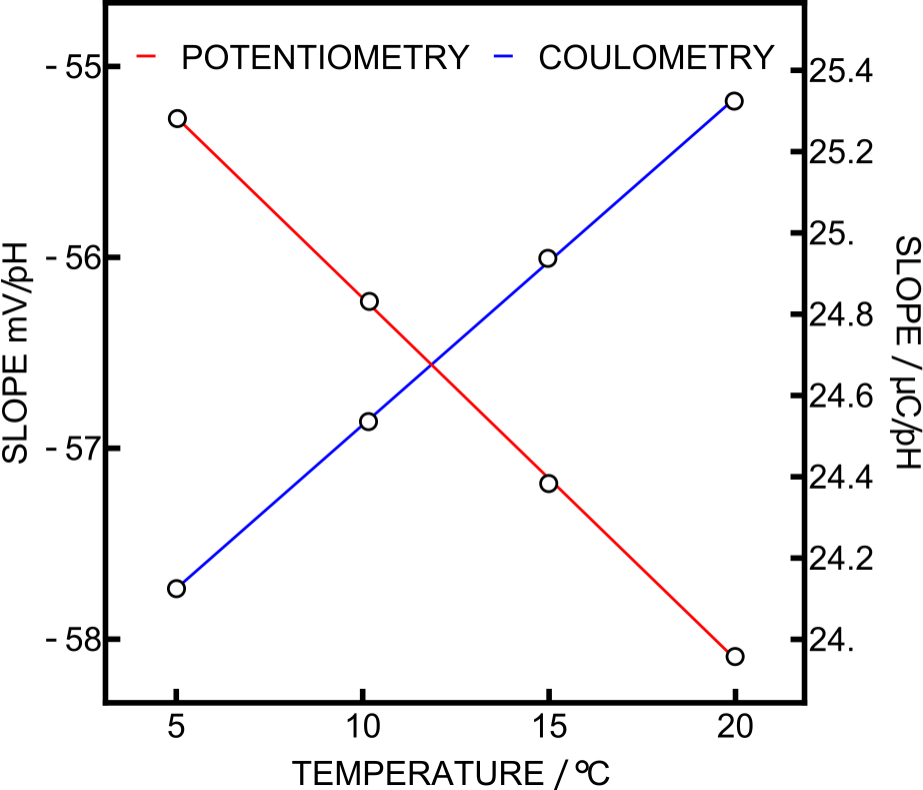
Experimental slopes obtained at different temperatures for both potentiometry and coulometry. Both lines are a linear fit of the experimental points.

A stability study over 20 h was performed in the laboratory at 15 °C by flowing Tris-stabilized synthetic seawater every 30 min (Figure 6), without any intermittent recalibration. The precision obtained during this experiment was 2 mpH for both readouts. This decreased value compared to the previous experiment certainly comes from sensor drift and dropout points, which may have been caused by the ingress of one or more small air bubbles owing to the probe being out of the water. This underlines the importance of a recalibration protocol in situ, as discussed below.

**Figure 6:**
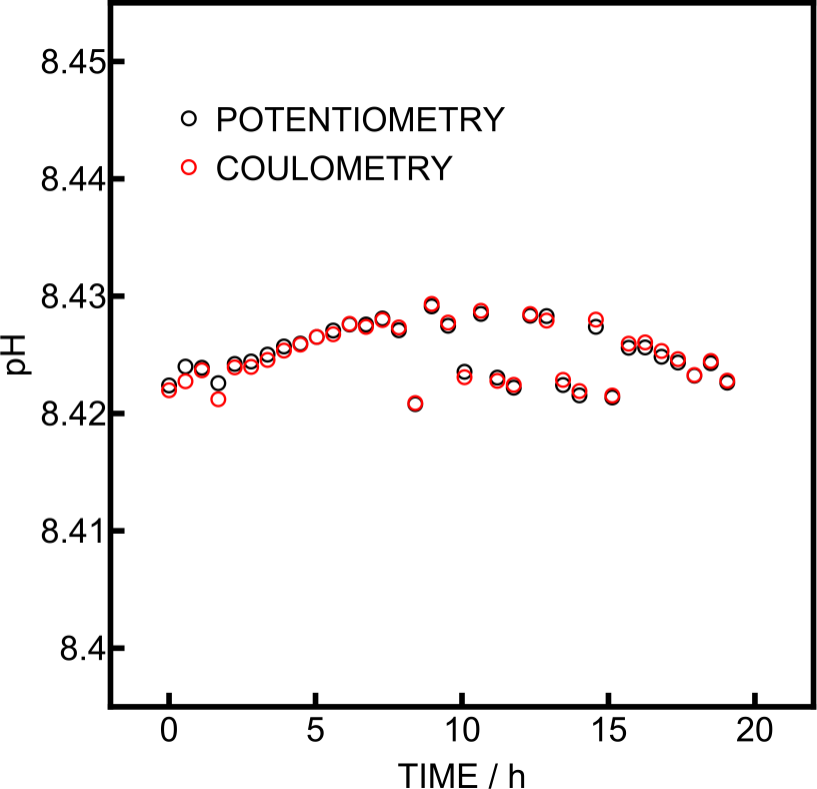
Laboratory pH measurement in Tris-stabilized synthetic seawater at 15 °C over 20 h.

### Field Application in the Krka River Estuary

The vertically stratified environment of the Krka River Estuary (Figure S6) offers attractive experimental conditions, allowing one to assess both the precision and accuracy of the submersible Couloprobe (Figure S6). On one hand, the well buffered bottom seawater layer offers an ideal environment to evaluate the precision of the sensor in situ because the pH is known not to change appreciably over time.^39^ On the other hand, the stratified nature with the sharp halocline (Figure S6) provides challenging conditions for electrochemical pH probes because the salinity gradient may negatively affect liquid junction potentials found in routine multiparameter pH probes, leading to inaccuracies (Figure S6b).^24^ Renewable and open-junction design such as the one used in this work are expected to reduce this source of inaccuracy.^24–26^

Data from a routine EXO2 multiparameter probe immersed in the seawater layer with a salinity of 38 over 24 h is presented in Figure 7a. The signal is stable at pH = 8.216 with a precision of 0.005 pH over the last 19 h. The latter mainly originates from the limited instrument resolution of 0.01 pH unit. This confirms that the signal from the Couloprobe should remain indifferent during the same time frame when immersed in the same seawater layer. The Couloprobe was immersed for 24 h at a depth of 6 m with a salinity of 37. The pH was measured by potentiometry and coulometry every 30 min (Figure 7b). As with the EXO2 multiparameter probe, an increase of 0.01 pH unit was observed during the first 5 h. These points were therefore not included in the precision evaluation. Potentiometry gave a pH of 8.220 and coulometry a pH of 8.225 over the last 19 h. The average signal repeatability was 0.0004 pH unit and 0.0005 pH unit, respectively. In both cases, the precision was 0.001 pH unit, which is excellent. The deviation between coulometry and potentiometry is thought to originate from the electronics of the probe that was subject to changes from the original electronic plans.

**Figure 7:**
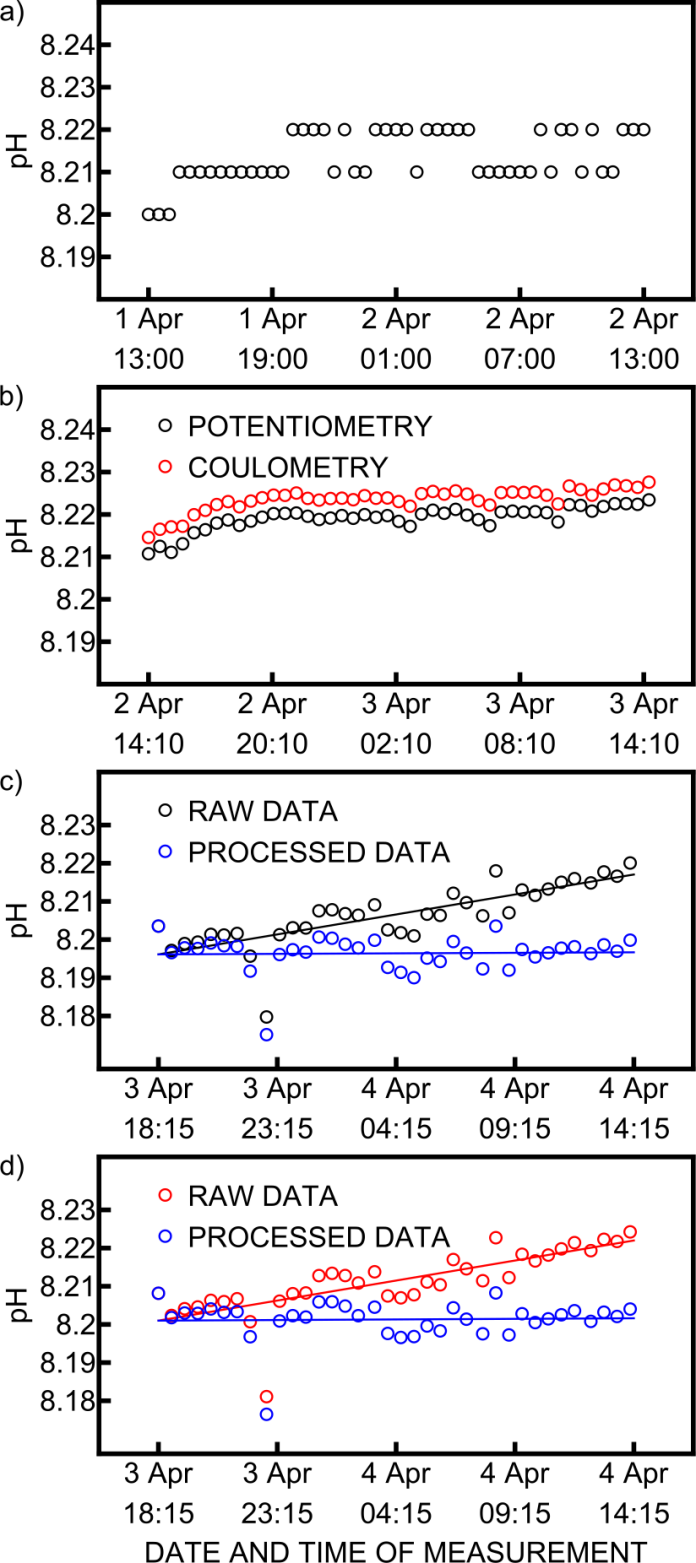
(a) pH measured with the EXO2 multiparameter probe in the seawater layer over 24 h. (b) pH measured by the Couloprobe in the seawater layer over 24 h. pH measured by (c) potentiometry and (d) coulometry with the Couloprobe in the seawater layer over 20 h. Synthetic seawater pH was measured every 4 h in (c) and (d) to allow for an in situ drift compensation, but only seawater pH measurements are displayed in this figure.

The potential of glass electrodes is known to drift over time.^14^ Therefore, the design of the probe also allows one to intermittently introduce a calibrant solution during long-term measurements. The chosen recalibration solution was Tris-stabilized synthetic seawater prepared according to previously published procedures.^36^ Synthetic seawater was preferred over NIST borax as a calibrant solution because its composition more closely matches the target sample. The recalibration procedure was evaluated by performing pH measurements in the seawater layer every 30 min for 20 h, with a recalibration measurement performed every 4 h to compensate for any drift. The synthetic seawater data was fitted with a linear function to obtain its drift in units of pH/h. The latter was then subtracted at each time from the seawater measurement to cancel out the drift. As the temperature did not change during the in situ experiment, the pH of the synthetic seawater was assumed to be constant during this experiment. The results for potentiometry and coulometry are presented in Figure 7c,d, respectively. The raw data drift is 1 mpH/h in both cases, which is excessive for a high-precision pH measurement over time. However, the synthetic seawater pH exhibited a very similar drift behavior than the pH of the seawater and canceled out the electrode drift when subtracted from the raw data (Figure 7c,d, blue dots). This demonstrated that the recalibration procedure is efficient to compensate for undesired drift, resulting in a precision of 5 mpH in this experiment. The average signal repeatability was 0.0007 pH unit for potentiometry and 0.0008 pH unit for coulometry. With the reagent bags used in this study (0.5 L), the Couloprobe may perform a maximum of 200 measurements. For the measurement frequency used in this work (every 30 min), this would represent 4 days of continuous monitoring.

As it was previously reported that ionic strength changes cause accuracy uncertainties,^14^ a depth profile experiment was carried out on April 7, 2025, to test the capability of the Couloprobe to handle salinity gradients present in the stratified Krka River Estuary. An EXO2 multiparameter probe was attached to the titanium cage with the sensing head next to the sample entrance of the Couloprobe for direct pH measurement comparison (Figure S7). The EXO2 pH probe was calibrated with the same buffers as the Couloprobe for consistency. The salinity recorded with the EXO2 probe was used to position the two sensors at a given depth to cover the entire salinity range (Figure 8a). The EXO2 probe was programmed to perform a measurement every 2 min, and the measurement closest in time of the coulometric measurement was extracted for comparison (Figure 8b). The Couloprobe was used to perform 4 measurements at each depth. However, as the halocline is highly dynamic, each measurement is treated individually. The second potentiometric and coulometric measurements are presented in Figure 8c and showed again an excellent correlation between each other. The average signal repeatability over the whole profile was 0.0004 pH unit for potentiometry and 0.0005 pH unit for coulometry. When comparing both probes, the observed pH change trends with depth are similar. However, an offset of about 0.1 pH unit was observed. This is likely due to the different RE configurations. In fact, the EXO2 probe uses an internal reference element (Ag/AgCl element in 3 M KCl with a fiber junction), while the Couloprobe implements open junctions. Moreover, the accuracy stated by the EXO2 manufacturer is ±0.1 pH unit. Another depth profile was performed with an OS316-*Plus* multiparameter probe (Figure S8). It is equipped with the same pH electrode as the Couloprobe and includes an external RE with a capillary as the liquid junction. The pH values were closer to the potentiometric pH of from the Couloprobe (Figure S8, black dots). In seawater, the pH difference between probes was only 0.016 pH unit. Unfortunately, the profile could not be done with the OS316-Plus multiparameter probe as a comparison because the conductivity sensor stopped working during the field campaign. As the halocline between brackish water and seawater is very dynamic, the probe is expected to experience different salinities over time at a given depth. The pH measurements in the halocline at different times are shown in Figure 8d–f. The pH is influenced by salinity change, and pH variations sometimes as low as 0.001 and 0.002 pH units were recorded. The upper and lower limits of the halocline are more subject to pH and salinity variations, while the middle of the halocline has a pH stable within ±0.005 pH.

**Figure 8:**
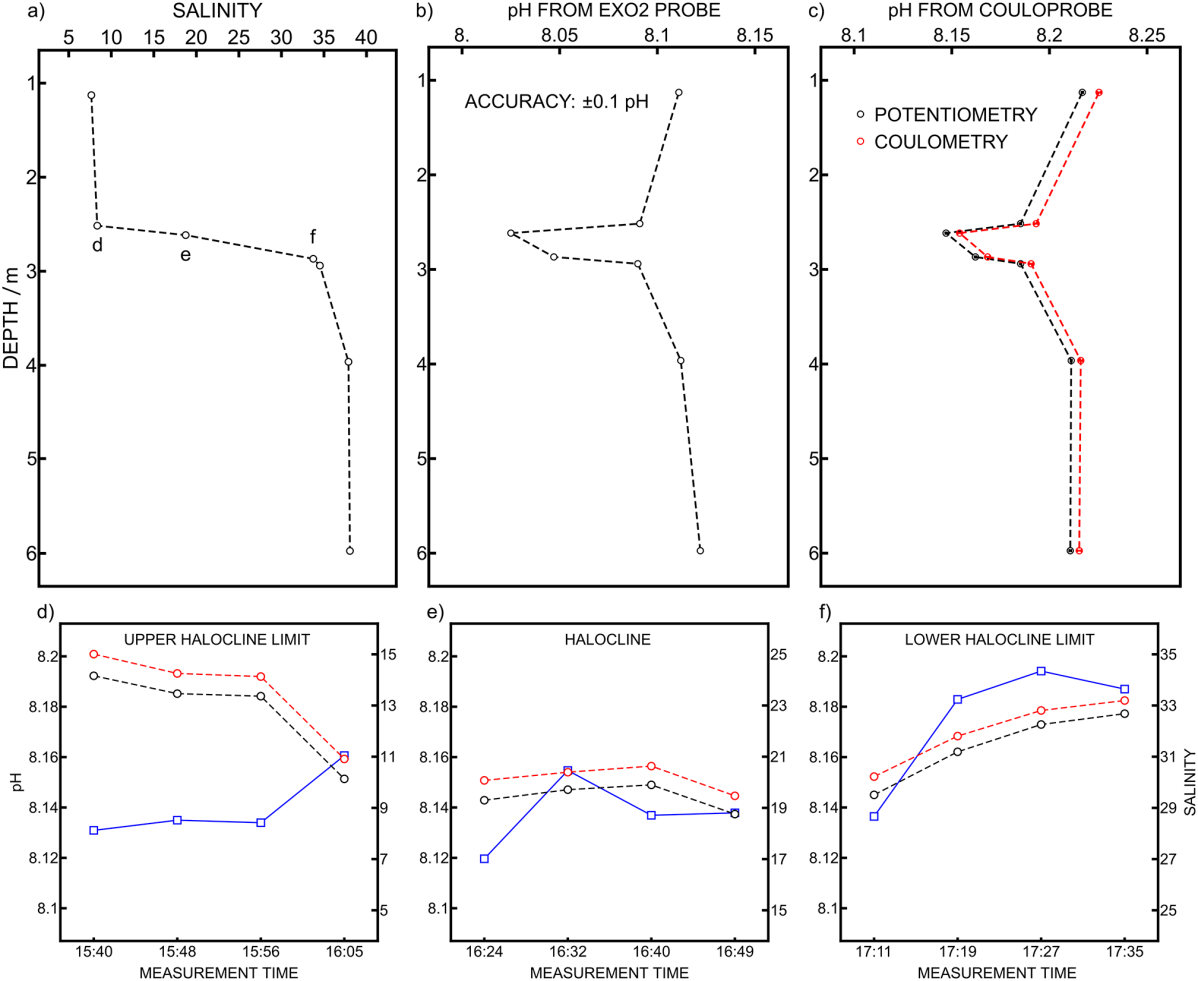
Depth profiles were measured on the Martinska study site. (a) Salinity from the EXO2 multiparameter probe, (b) pH from the EXO2 multiparameter probe, (c) pH from the Couloprobe with potentiometric and coulometric readouts. (d–f) Coulometric pH (red circles), potentiometric pH (black circles), and salinity (blue squares) measured in the dynamic halocline between brackish water and seawater. The corresponding depths are indicated in (a).

## Conclusions

A high precision submersible pH electrochemical probe based on constant potential coulometry was developed, characterized, and deployed in situ in the Krka River Estuary, Croatia. The behavior of the electronic circuit approached ideality, even when changing the measurement temperature. Long-term measurements allowed to evaluate the instrument precision. Recalibration with synthetic seawater pH was also successfully implemented to cancel out uncompensated drift during long-term measurements. A pH depth profile investigated the behavior of the probe with changing salinity due to stratification.

Overall, the Couloprobe performs better in terms of precision than the commercially available EXO2 multiparameter probe. This is not surprising since multiparameter probes are built for convenience, and their focus is to provide a range of parameters at high sampling frequency. For this reason, they tend to lack accuracy and have only limited precision. Accuracy was improved in the Couloprobe by implementing chemical symmetry and open junctions. A remaining limitation is that the uncertainty of the reference buffer must be considered, which places increased demands on the availability of reference buffers with exactly known pH values and temperature behavior.

Another conclusion of this work is that coulometry and potentiometry perform in a similar manner in terms of precision in this particular study. For the coulometric readout to clearly outperform potentiometry, sensor drift must be even better controlled. Surprisingly, the two readouts are separated by an offset that corresponds to 0.3 mV. As the chemistry of the glass electrode dictates both the potentiometric and the coulometric responses, the offset likely originates from the electronics of the probe. The latter was subject to multiple modifications during development, which might influence the coulometric protocol. A revised version is expected to bring the two readouts closer together. This probe design achieves precision on the order of 1 mpH, thereby approaching that of indicator-based spectrophotometry with the advantage of fewer systematic errors.

Further work with this setup should aim to assess the liquid junction potential uncertainty and compare the pH values obtained with the Couloprobe with optical pH measurements on various pH scales.

## Supplementary Material



## Notes

Pictures of the electronic board, calibration with an external source meter, chloride calibration, pH calibration, experimental zero-point pH, fitting coefficients for both slopes and zero-point pH with temperature, error propagation procedure, depth profiles for temperature, salinity and pH, picture of the EXO2 multiparameter probe mounted on the Couloprobe and detailed electronic schemes. (PDF)
