# Supplementary material for: Symmetrical pH Electrochemical Cell Coupled to Constant Potential Coulometry for Improved Sensitivity and Precision: Part 2. Submersible Probe for In Situ Measurements
Source: ACS Meas Sci Au. 2026 Feb 10;6(2):497–506. doi: 10.1021/acsmeasuresciau.5c00198 (PMC13087930; doi:10.1021/acsmeasuresciau.5c00198)
Supplement: Supplementary file 1 [file tg5c00198_si_001.pdf]

Supporting Information for:

Symmetrical pH Electrochemical Cell Coupled to Constant Potential Coulometry for Improved Sensitivity and Precision: Part 2. Submersible Probe for In Situ Measurements

Robin Nussbaum[1], Stéphane Jeanneret[1], Thomas Cherubini[1], Mary-Lou Tercier-Waeber[1], Dario Omanovic[2] and Eric Bakker[1]*

[1] Department of Inorganic and Analytical Chemistry, University of Geneva, Quai Ernest-Ansermet 30, 1211 Geneva, Switzerland.

[2] Laboratory for Physical Chemistry of Traces. Center for Marine and Environmental Research, Ruđer Bošković Institute, POB 180, 10002 Zagreb, Croatia.

*Eric.Bakker@unige.ch

SUPPORTING FIGURES

Figure S1. a) top, b) bottom, c) side views of the electronic board. 2
Figure S2. a) Potentiometric and b) coulometric calibrations of the electronic circuit when imposing an external voltage with a source meter. The red line is the linear fit of the points, while the blue line is a theoretical fit obtained by converting the voltages in a) into charges. 2
Figure S3. Coulometric chloride calibration. Experimental slopes: -24.3 μC, corresponding to -55.5 mV. Theoretical slopes: -25.5 μC, corresponding to -58.2 mV. 3
Figure S4. Coulometric pH calibration over a wide pH range. Experimental slope: 25.4 μC, corresponding to -58.0 mV. Theoretical slopes: -25.5 μC, corresponding to -58.2 mV. 3
Figure S5. pH of the borax reference buffer determined experimentally at different temperatures in a) potentiometry and b) coulometry. The fitting coefficients are displayed in Table S1. 3
Figure S6. Depth profiles of a) temperature, b) salinity and c) pH, recorded with an EXO2 multiparameter probe on the Martinska study site. 5
Figure S7. Picture of Couloprobe with an EXO2 multiparameter probe (blue tube) attached on it before the depth profile experiment. 5
Figure S8. Comparison between pH depth profile obtained with the OS316-*Plus* multiparameter probe (blue dashed line) and potentiometric pH from the Couloprobe (black dots). 6
Figure S9. Detailed scheme of the electronic board (part 1). 7
Figure S10. Detailed scheme of the electronic board (part 2). 8
Figure S11. Detailed scheme of the electronic board (part 3). 9
Figure S12. Detailed scheme of the electronic board (part 4). 10
Figure S13. Detailed scheme of the electronic board (part 5). 11
Figure S14. Detailed scheme of the electronic board (part 6). 12
Figure S15. Detailed scheme of the electronic board (part 7). 13

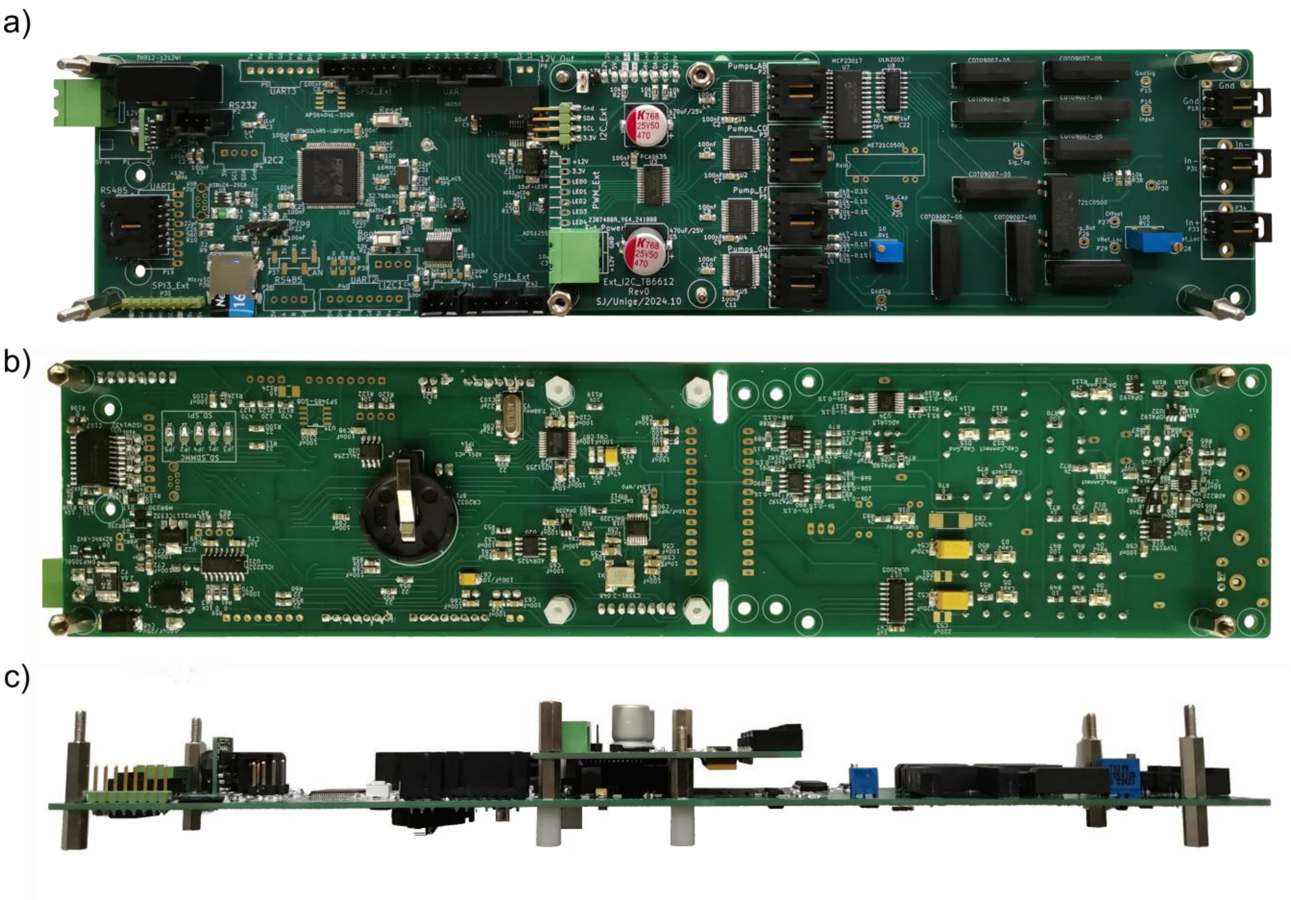

Figure S1. a) top, b) bottom, c) side views of the electronic board.

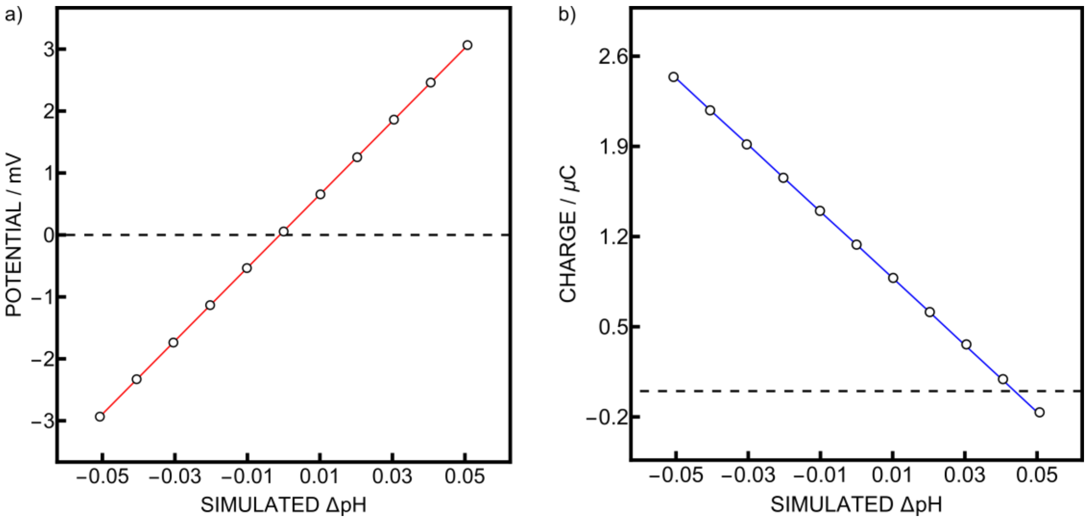

Figure S2. a) Potentiometric and b) coulometric calibrations of the electronic circuit when imposing an external voltage with a source meter. The red line is the linear fit of the points, while the blue line is a theoretical fit obtained by converting the voltages in a) into charges.

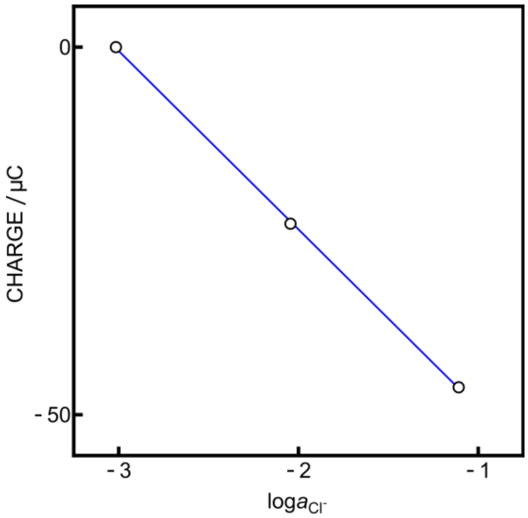

Figure S3. Coulometric chloride calibration. Experimental slopes: -24.3 µC, corresponding to -55.5 mV. Theoretical slopes: -25.5 µC, corresponding to -58.2 mV.

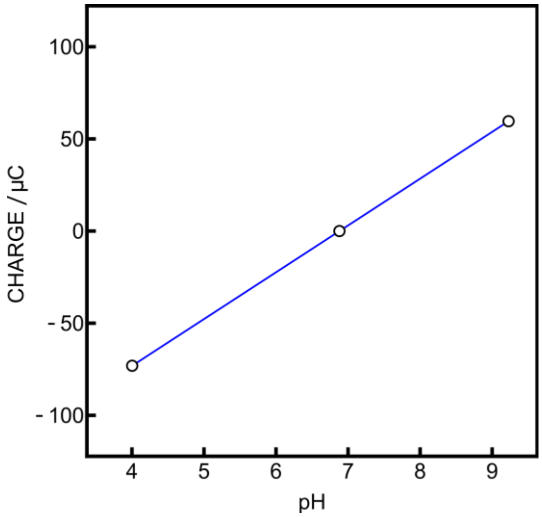

Figure S4. Coulometric pH calibration over a wide pH range. Experimental slope: 25.4 µC, corresponding to -58.0 mV. Theoretical slopes: -25.5 µC, corresponding to -58.2 mV.

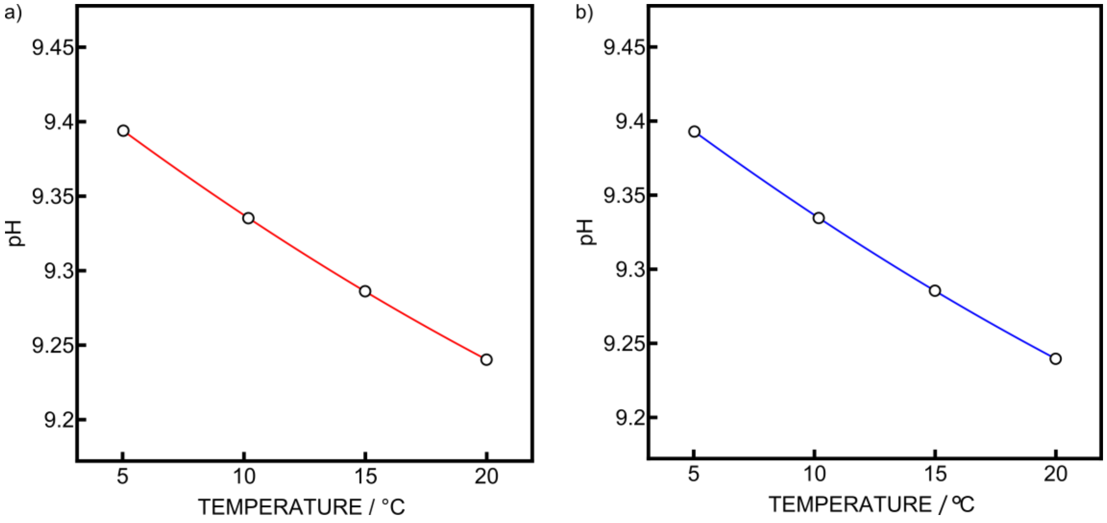

Figure S5. pH of the borax reference buffer determined experimentally at different temperatures in a) potentiometry and b) coulometry. The fitting coefficients are displayed in Table S1.

Fit	Readout	a	b	c
Slope	Potentiometry	-54.32 ± 0.03	-0.189 ± 0.002	-
	Coulometry	23.72 ± 0.02	0.0805 ± 0.0009	-
pH_0	Potentiometry	9.4569 ± 0.0005	-0.01301 ± 0.00010	0.000112 ± 0.000004
	Coulometry	9.4553 ± 0.0003	-0.01296 ± 0.00006	0.000108 ± 0.000002

Table S1. Fit parameters for temperature correction of slope (a+bx) and pH_0 (a+bx+cx2) for potentiometric and coulometric readout.

Procedure to convert a potential difference or charge to pH value considering the temperature measurement error.

This example is given for the charge but an analogous procedure was done for the potential difference.

The slope s versus temperature T was fitted with the following equation:

$$s = a + bT \tag{ES1}$$

where a and b are fitting parameters reported in Table S1 and T the temperature. The error of the slope is given by:

$$\delta_{\text{slope}}^2 = \nabla s^T \cdot \Sigma \cdot \nabla s + \left(\frac{\partial s}{\partial T}\right)^2 \tag{ES2}$$

The pH_0 (expressed as fit parameter f) versus temperature was fitted with the following equation:

$$f = a + bT + cT^2 \tag{ES3}$$

where a, b and c are fitting parameters reported in Table S1. The error on the pH_0 is given by:

$$\delta_{\text{pH borax}}^2 = \left(\frac{\partial f}{\partial a}\right)^2 \delta_a^2 + \left(\frac{\partial f}{\partial b}\right)^2 \delta_b^2 + \left(\frac{\partial f}{\partial c}\right)^2 \delta_c^2 + \left(\frac{\partial f}{\partial T}\right)^2 \sigma_T^2 \tag{ES4}$$

The charge can be converted to pH with Equation (3) of the main text.
The conversion of the signal repeatability (standard deviation) expressed in coulombs into pH unit is achieved through:

$$\delta_{\text{pH sample}} = \sqrt{\left(\frac{1}{s}\delta_Q\right)^2 + \left(\frac{Q}{s^2}\delta_s\right)^2 + \left(\delta_{\text{pH borax}}\right)^2} \tag{ES5}$$

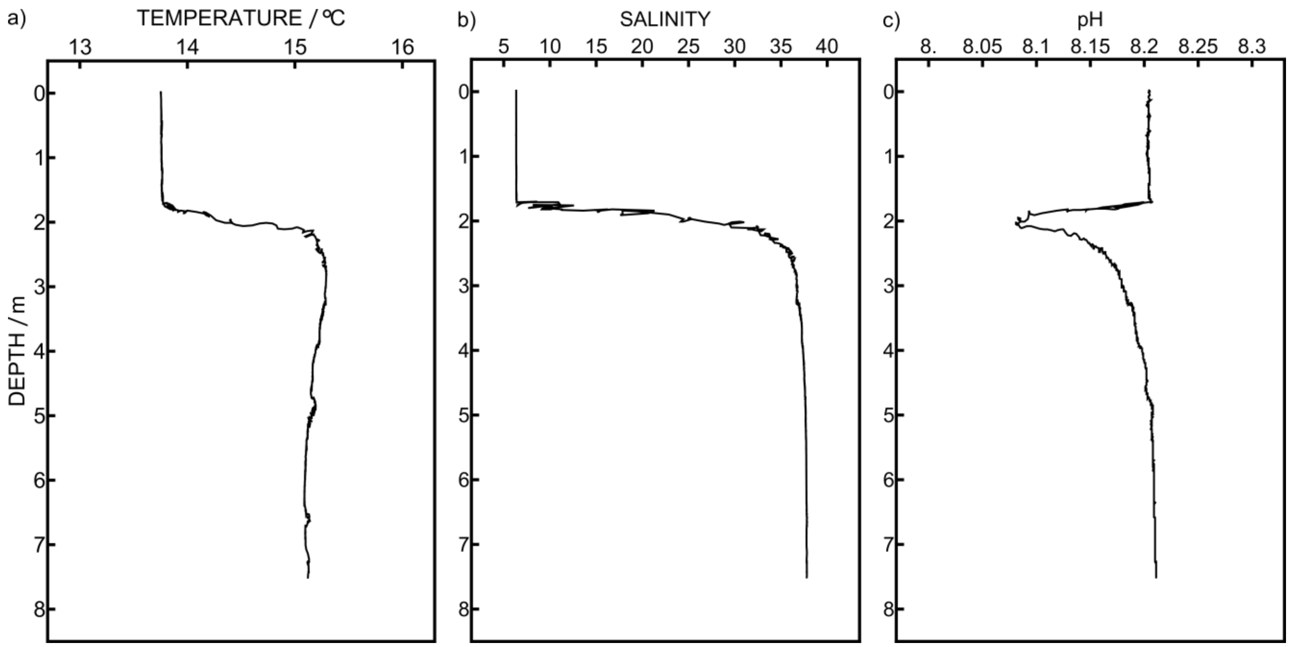

Figure S6. Depth profiles of a) temperature, b) salinity and c) pH, recorded with an EXO2 multiparameter probe on the Martinska study site.

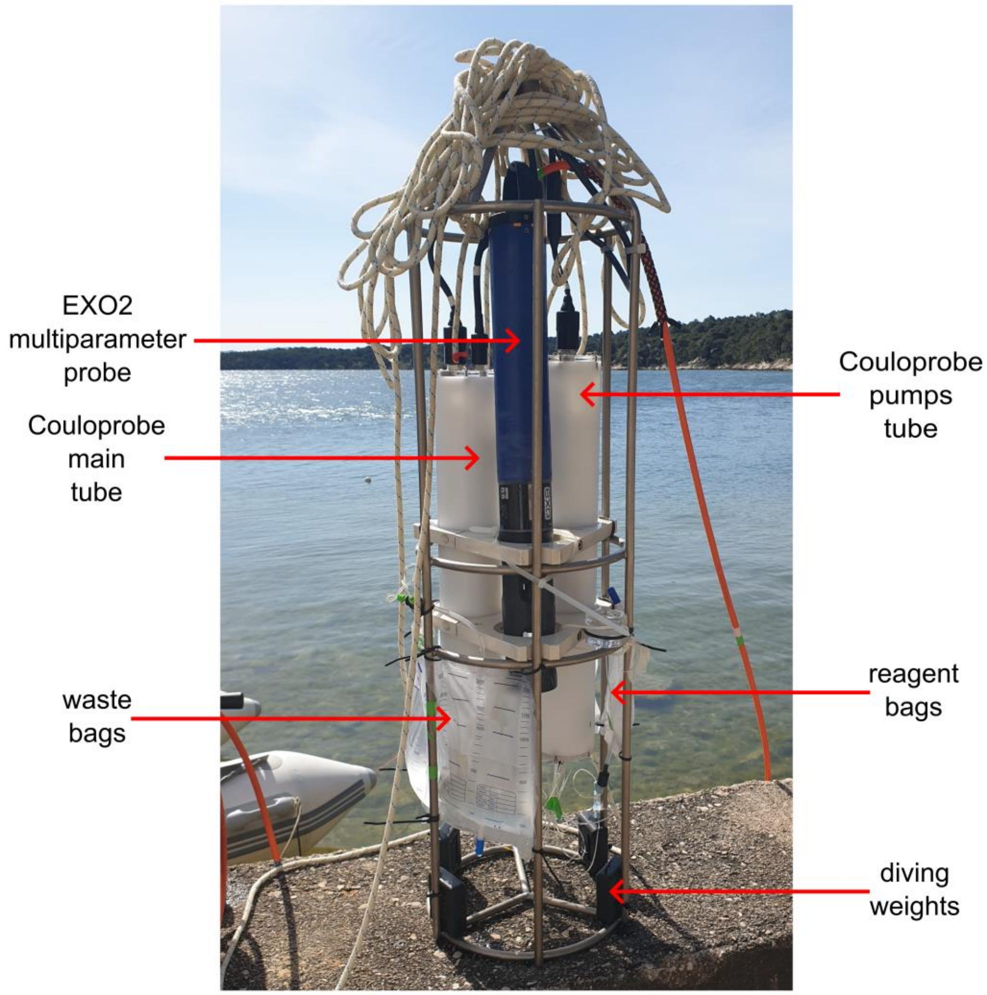

Figure S7. Picture of Couloprobe with an EXO2 multiparameter probe (blue tube) attached on it before the depth profile experiment.

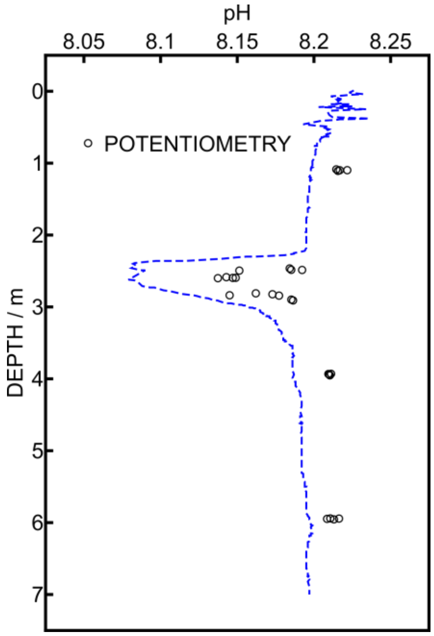

Figure S8. Comparison between pH depth profile obtained with the OS316-*Plus* multiparameter probe (blue dashed line) and potentiometric pH from the Couloprobe (black dots).

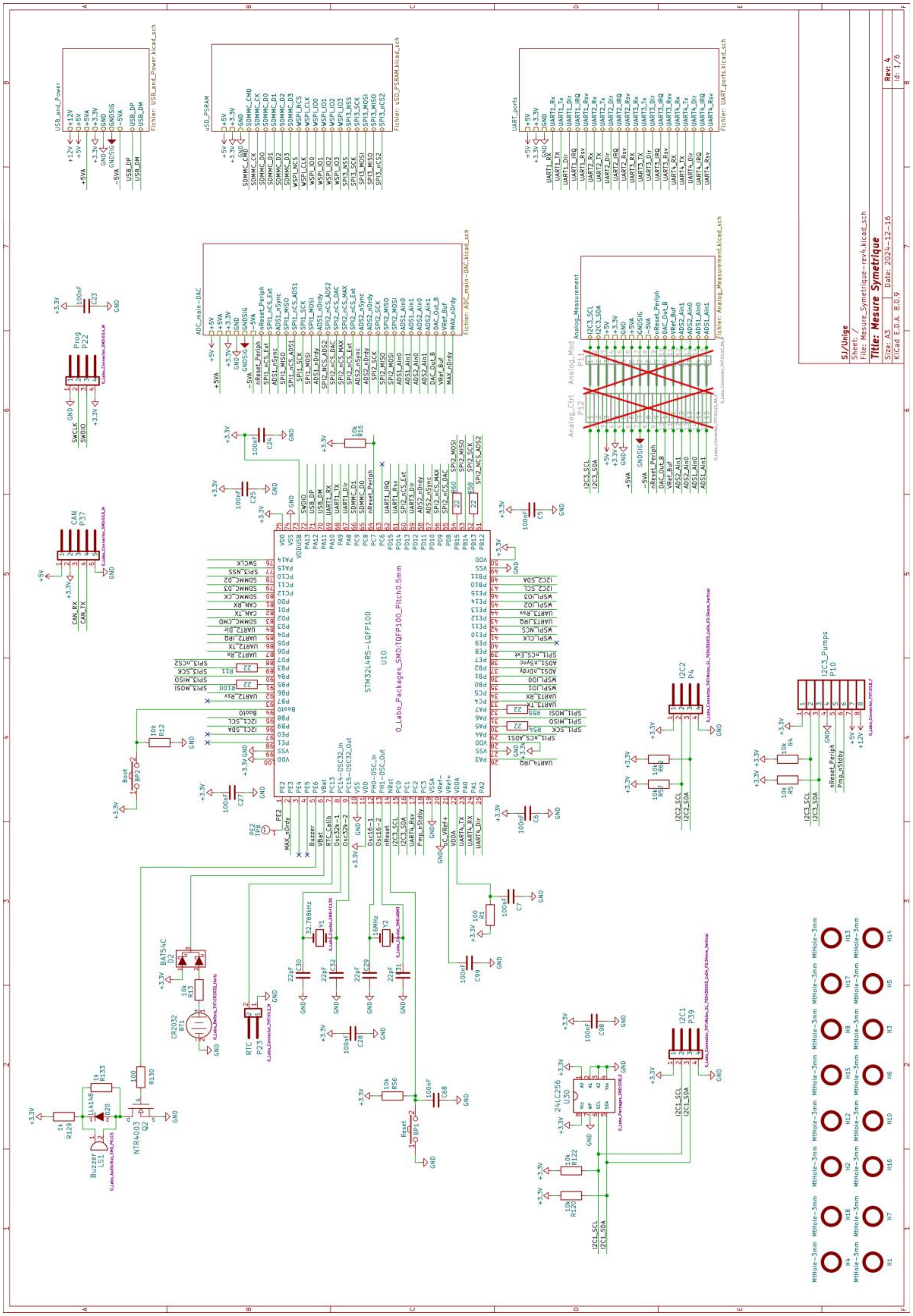

Figure S9. Detailed scheme of the electronic board (part 1).

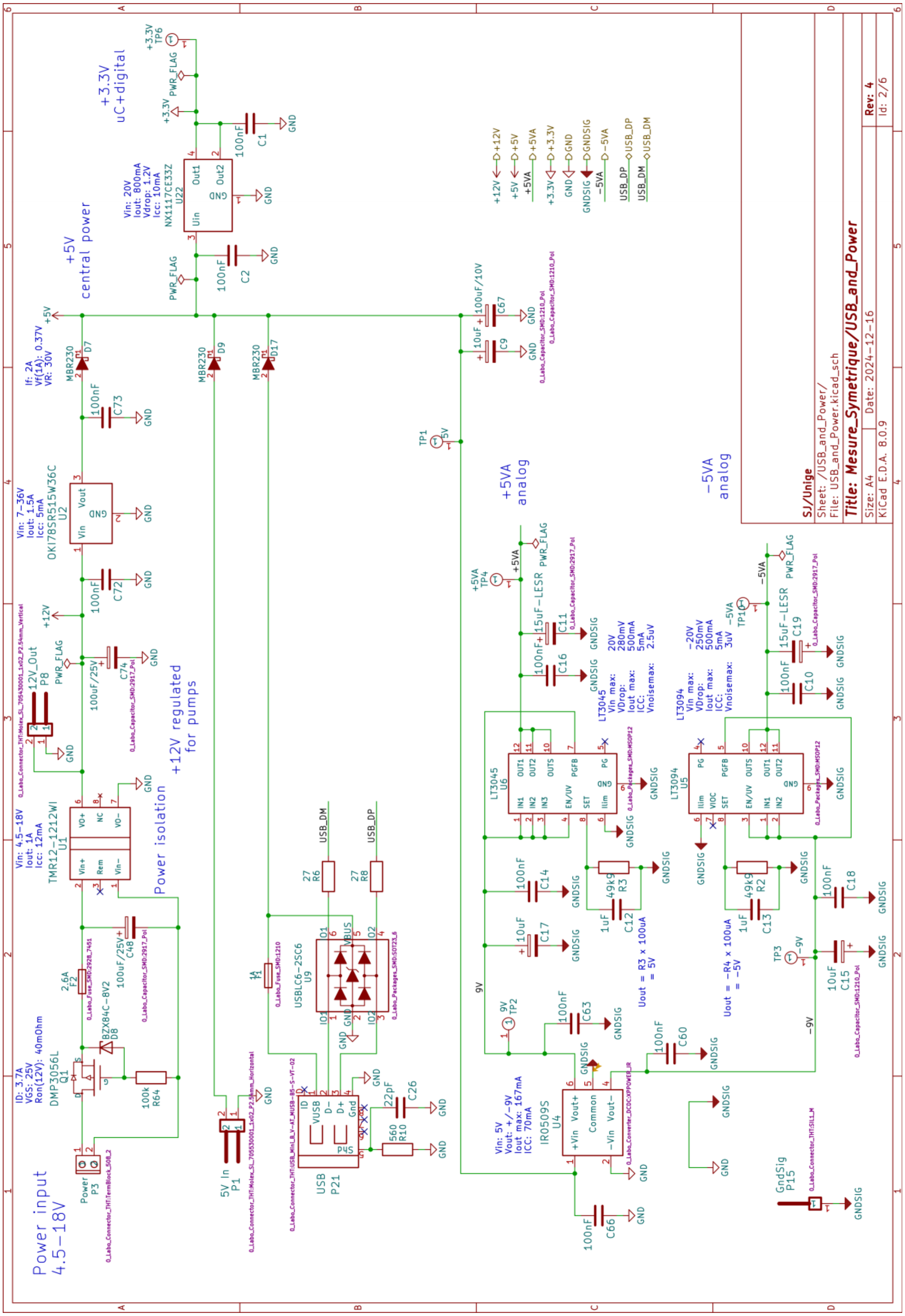

Figure S10. Detailed scheme of the electronic board (part 2).

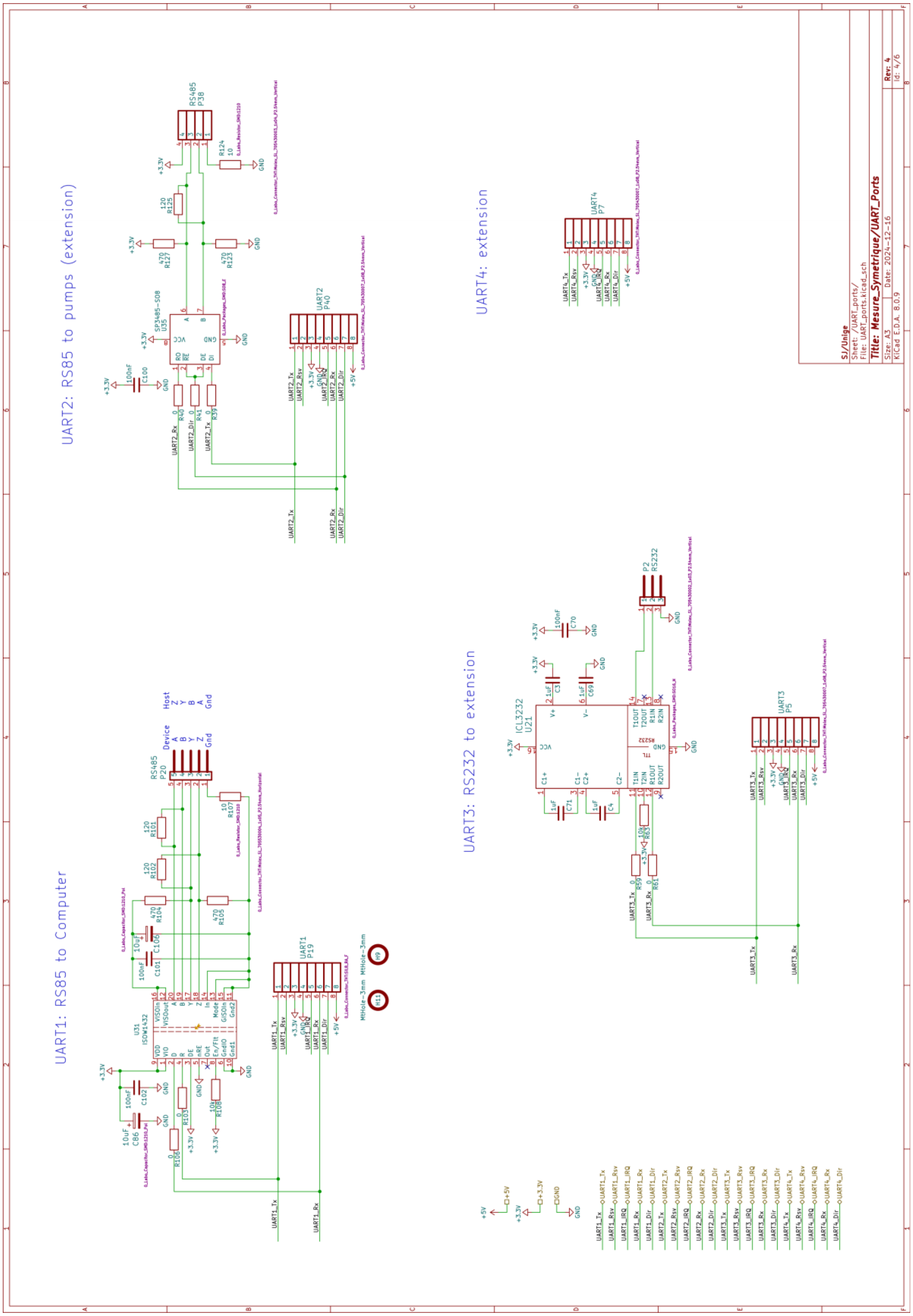

Figure S11. Detailed scheme of the electronic board (part 3).

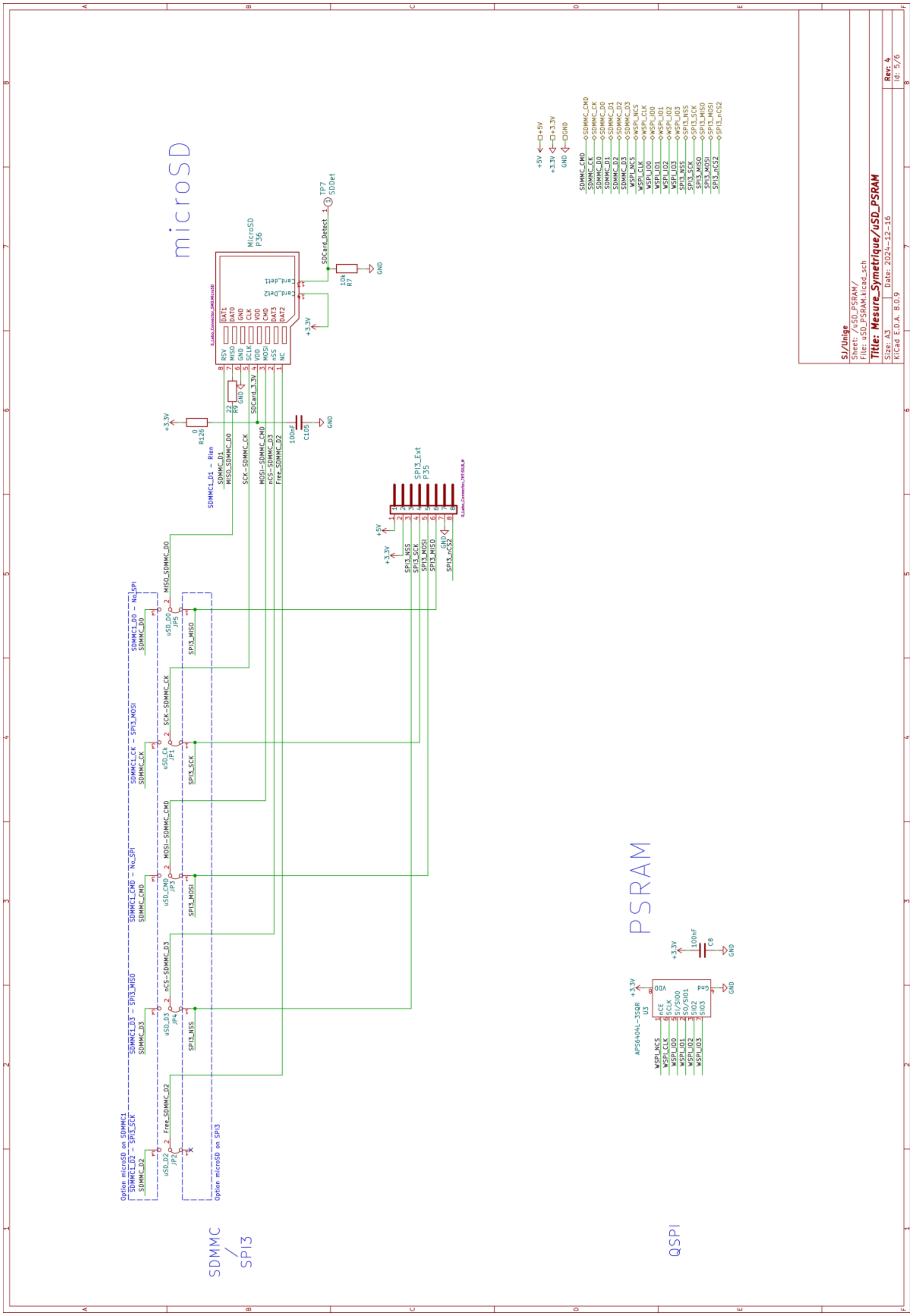

Figure S12. Detailed scheme of the electronic board (part 4).

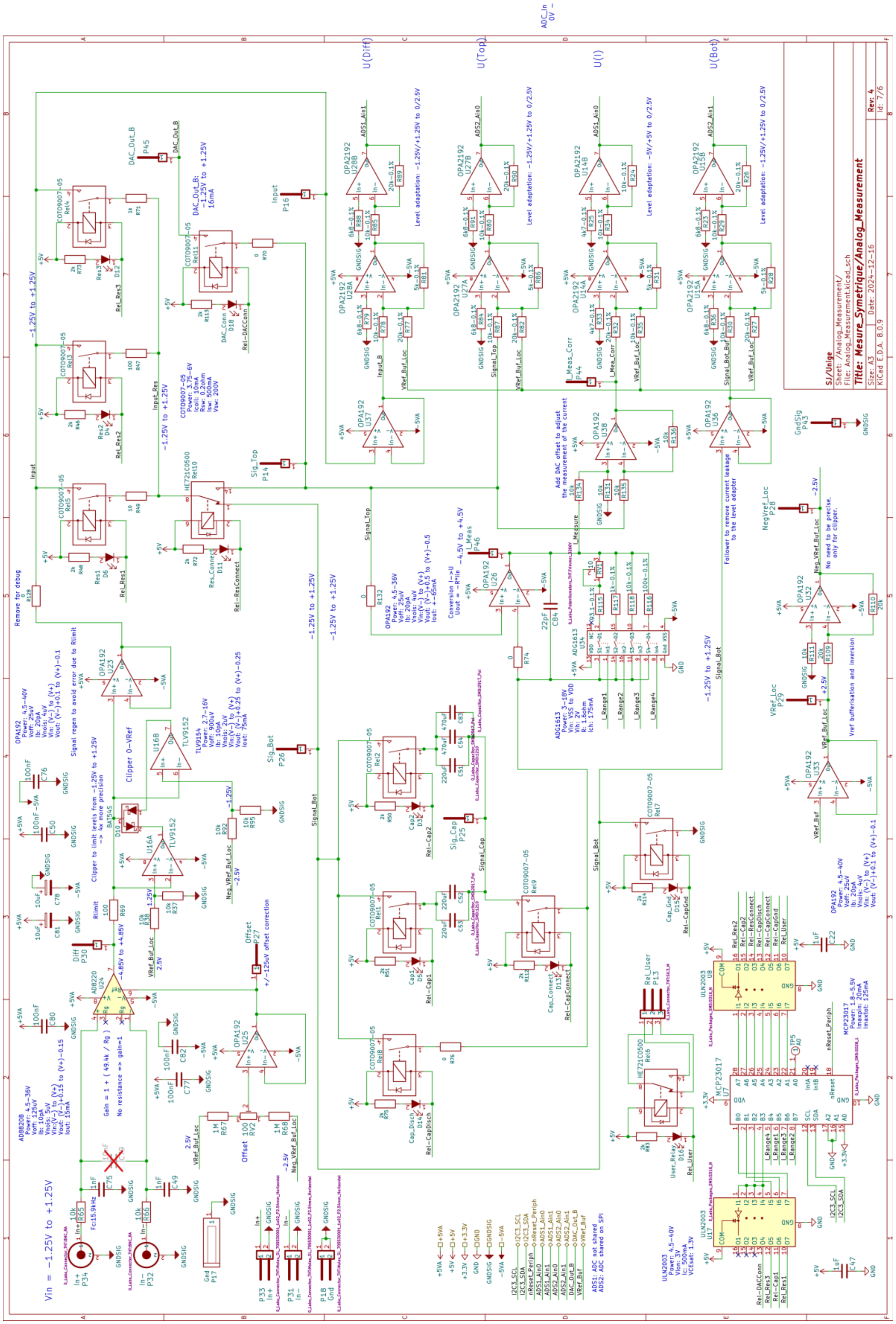

Figure S13. Detailed scheme of the electronic board (part 5).

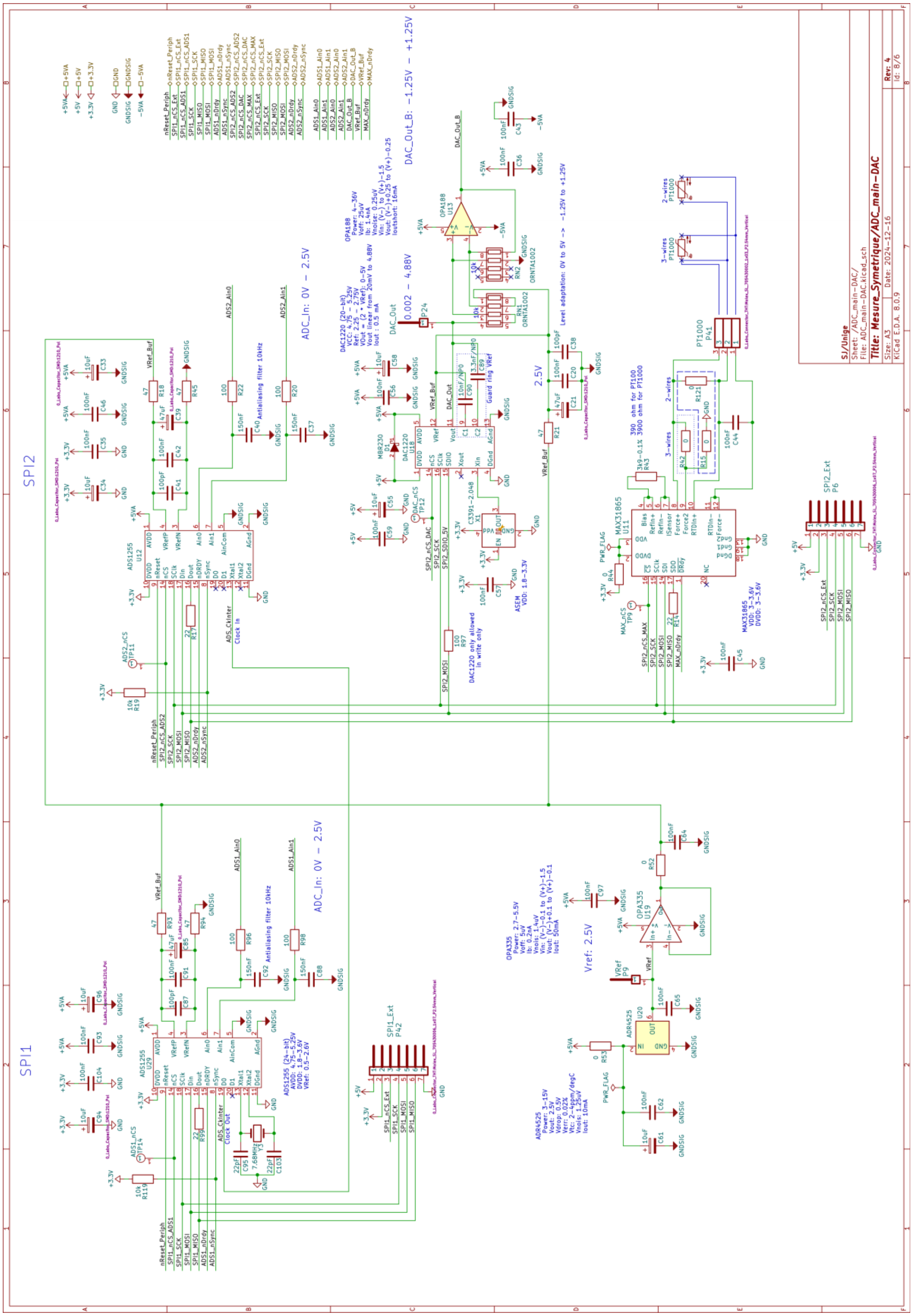

Figure S14. Detailed scheme of the electronic board (part 6).

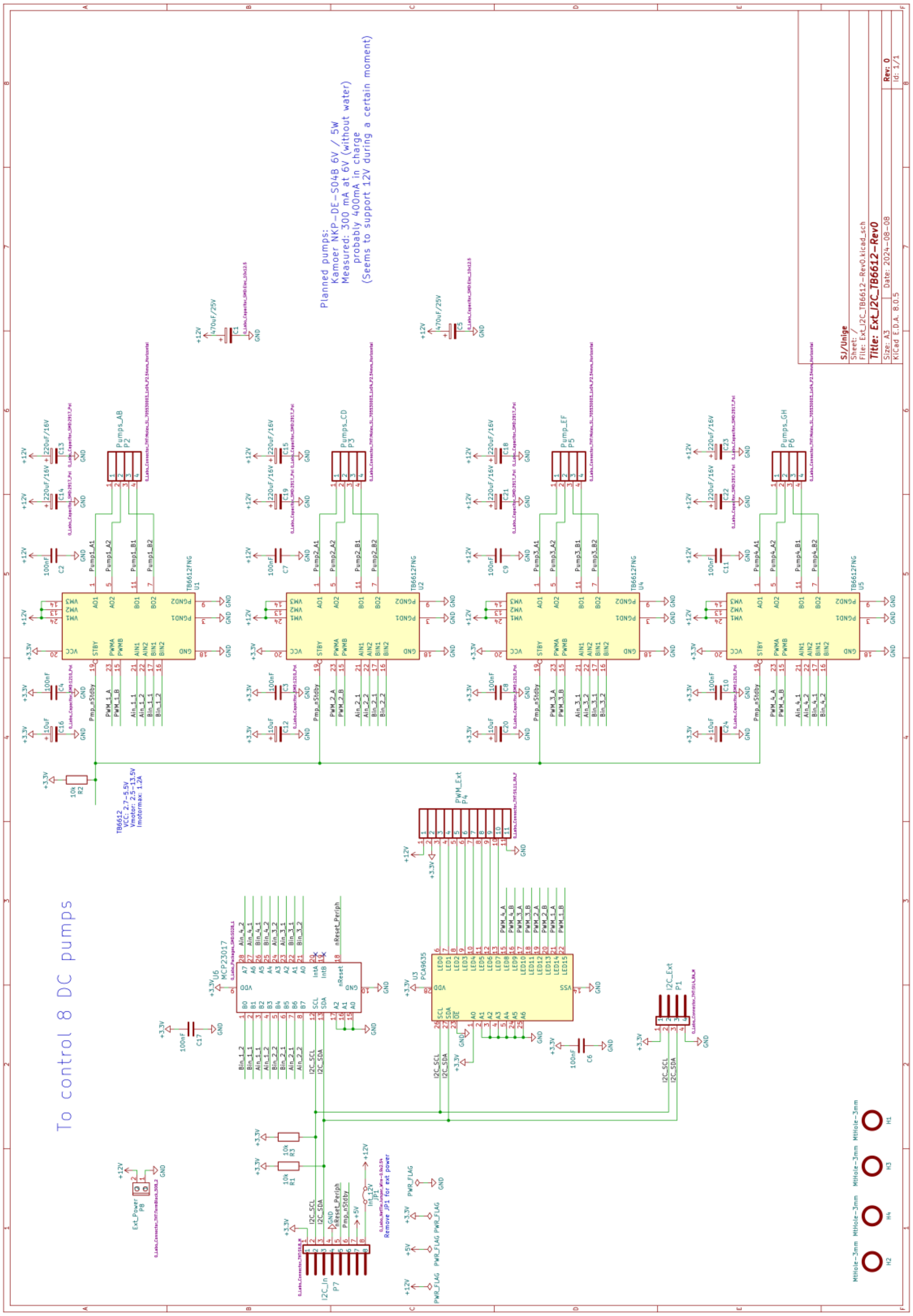

Figure S15. Detailed scheme of the electronic board (part 7).
